# Supplementary material for: Evaluation of Immunodiagnostic Performances of Neospora caninum Peroxiredoxin 2 (NcPrx2), Microneme 4 (NcMIC4), and Surface Antigen 1 (NcSAG1) Recombinant Proteins for Bovine Neosporosis
Source: Animals (Basel). 2024 Feb 6;14(4):531. doi: 10.3390/ani14040531 (PMC10885977; doi:10.3390/ani14040531)
Supplement: Supplementary file 1 [file animals-14-00531-s001.zip › Figure S1.docx]

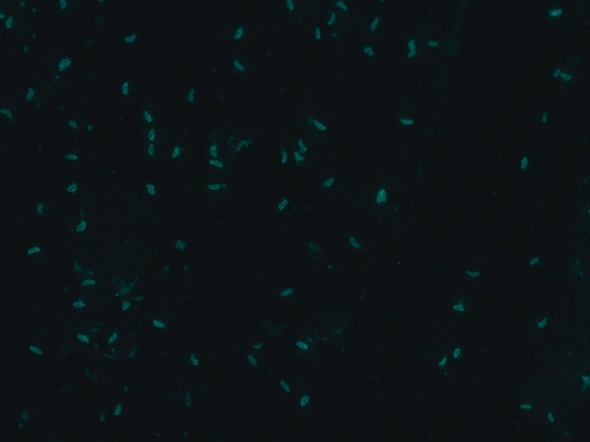

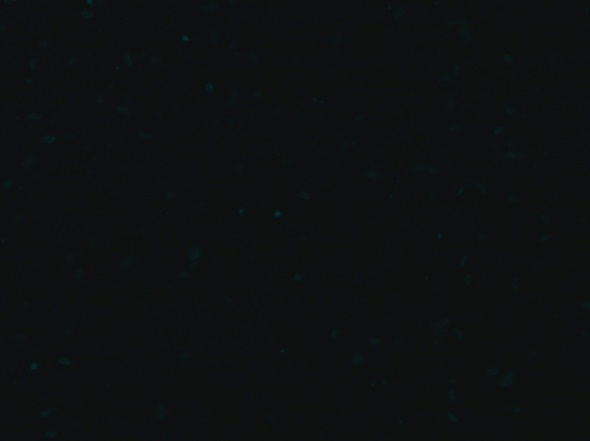


**B**

**A**

**Figure S1.** *N. caninum* probed with anti-*N. caninum* cattle serum, followed by fluorescent-labeled anti-bovine IgG, exhibited extensive peripheral fluorescein of *N. caninum* tachyzoites (A). In contrast, *N. caninum* probed with negative serum and then with fluorescent-labeled anti-bovine IgG displayed an absence of fluorescein signal (B).
