# Supplementary material for: Evaluation of Immunodiagnostic Performances of Neospora caninum Peroxiredoxin 2 (NcPrx2), Microneme 4 (NcMIC4), and Surface Antigen 1 (NcSAG1) Recombinant Proteins for Bovine Neosporosis
Source: Animals (Basel). 2024 Feb 6;14(4):531. doi: 10.3390/ani14040531 (PMC10885977; doi:10.3390/ani14040531)
Supplement: Supplementary file 1 [file animals-14-00531-s001.zip › Figure S2.docx]

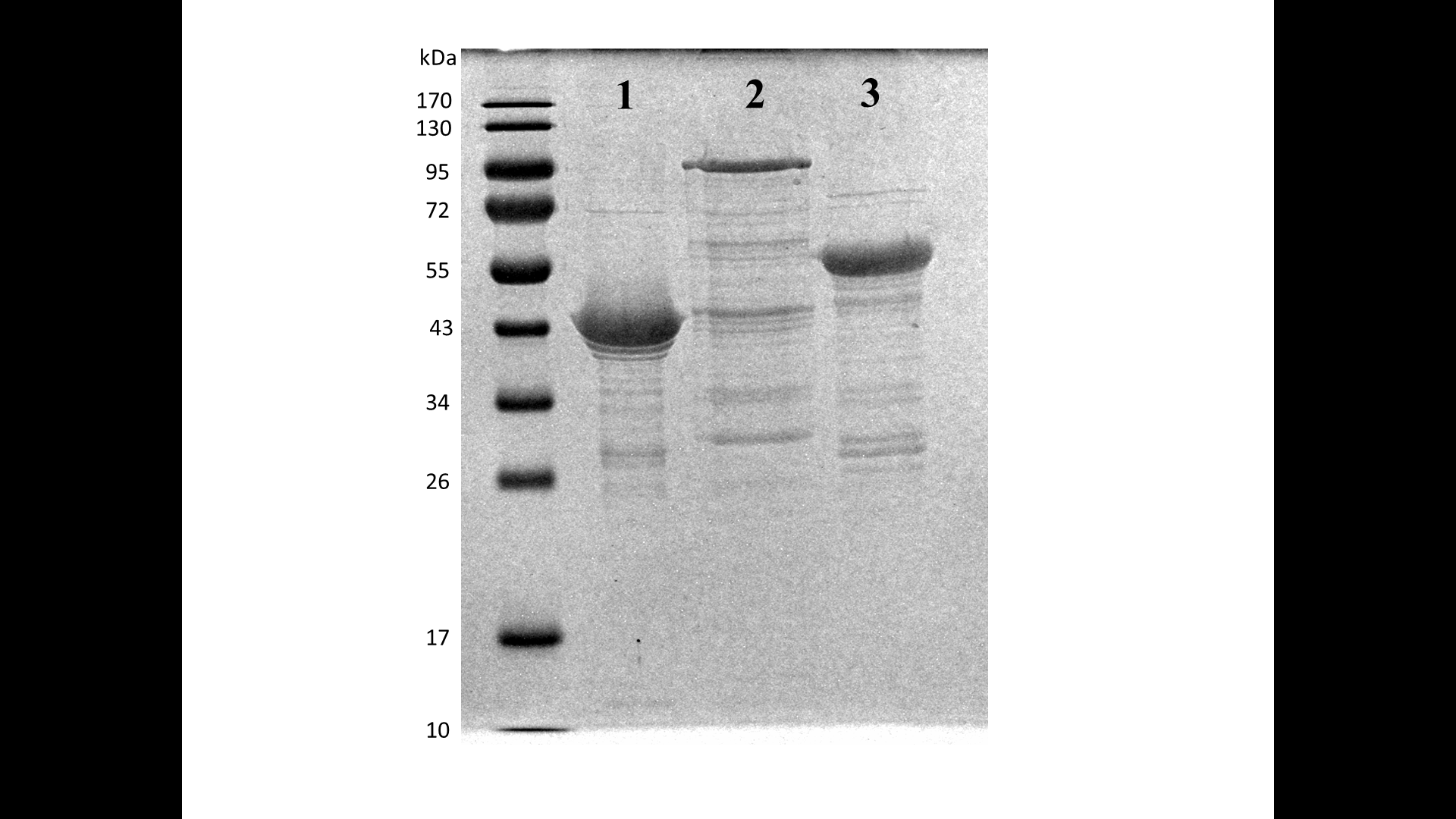


**Figure S2.** Coomassie blue stained SDS-PAGE (12% gel) of NcPrx2 (1), NcMIC4 (2) and NcSAG1 (3) recombinant proteins.

Abbreviations: SDS-PAGE, Sodium Dodecyl Sulphate-Polyacrylamide Gel Electrophoresis; NcPrx2-GST, *N. caninum*-derived peroxiredoxin 2; NcMIC4, *N. caninum* microneme 4; NcSAG1, *N. caninum* surface antigen 1; GST, Glutathione S-transferase (GST) tags.
