# Supplementary material for: Evaluation of Immunodiagnostic Performances of Neospora caninum Peroxiredoxin 2 (NcPrx2), Microneme 4 (NcMIC4), and Surface Antigen 1 (NcSAG1) Recombinant Proteins for Bovine Neosporosis
Source: Animals (Basel). 2024 Feb 6;14(4):531. doi: 10.3390/ani14040531 (PMC10885977; doi:10.3390/ani14040531)
Supplement: Supplementary file 1 [file animals-14-00531-s001.zip › Figure S4.docx]

**
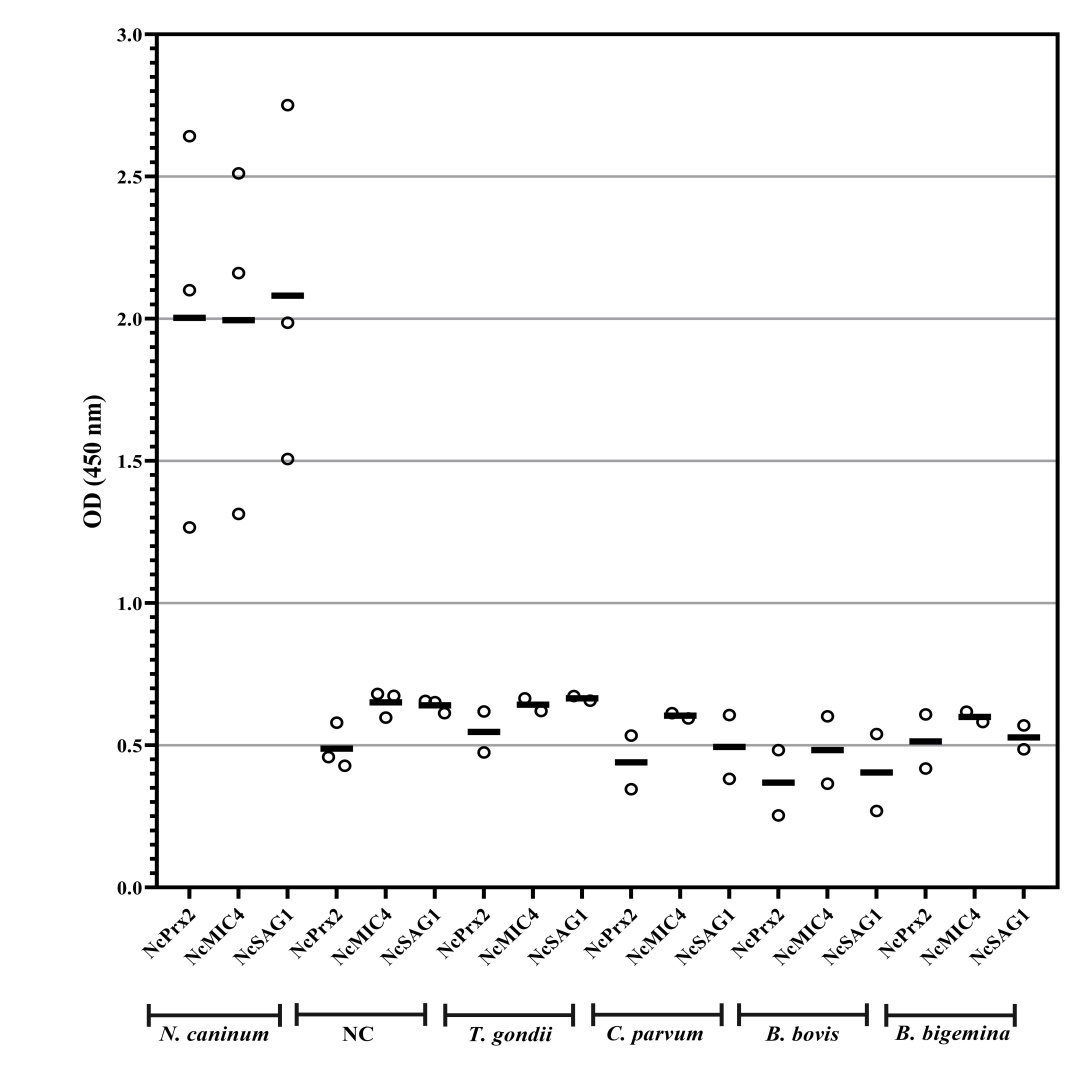
**

**Figure S4.** Evaluation of NcPrx2, NcMIC4 and NcSAG1 tested with known bovine infected and uninfected sera by indirect ELISA. Each bar represents the mean of the test sera (*N. caninum*, sera from infected *N. caninum*: N=3, NC, uninfected sera: N=3, and other sera from infected *Toxoplasma gondii*: N=2, *Cryptosporidium parvum*: N=2, *Babesia bovis*: N=2 and *Babesia bigemina*: N=2, respectively).

Abbreviations: NcPrx2 = *N. caninum*-derived peroxiredoxin 2; NcMIC4 = *N. caninum* microneme 4; NcSAG1 = *N. caninum* surface antigen 1; ELISA = enzyme-linked immunosorbent assay.
